# Association of Eating Behavior, Nutritional Risk, and Frailty with Sarcopenia in Taiwanese Rural Community-Dwelling Elders: A Cross-Sectional Study

**DOI:** 10.3390/nu14163254

**Published:** 2022-08-09

**Authors:** Ya-Wen Kuo, Chu-Wei Chen, Jia-Yu Zhang, Jiann-Der Lee

**Affiliations:** 1 Department of Neurology, Chiayi Chang Gung Memorial Hospital, No. 6, West Sec., Jiapu Road, Puzi City 613, Taiwan; 2 Department of Nursing, College of Nursing, Chang Gung University of Science and Technology, No. 2, Sec. W., Jiapu Rd., Puzi City 613, Taiwan; 3 The Third Research Division, Chung-Hua Institution for Economic Research, 75 Chang-Hsing Street, Taipei 10672, Taiwan; 4 Department of Nursing, Changhua Christian Hospital, No. 135, Nanxiao St., Changhua City 500, Taiwan; 5 College of Medicine, Chang Gung University, No. 259, Wenhua 1st Rd., Guishan Dist., Taoyuan 333, Taiwan

**Keywords:** eating behavior, nutritional risk, frailty, sarcopenia

## Abstract

This cross-sectional study assessed the association of eating behavior, nutritional risk, and frailty with sarcopenia in 208 community-dwelling individuals aged ≥65 years who were recruited from random rural community care centers in Chiayi County, Taiwan. The participants’ eating behavior was categorized into six categories. The gait speed (GS), grip strength, and appendicular skeletal muscle mass (ASM) were assessed based on these three parameters, which revealed that 50.9% of the participants had sarcopenia. In an adjusted model, water intake (odds ratio (OR) = 0.99, *p* = 0.044), dairy product intake (OR = 0.42, *p* = 0.049), body mass index (BMI) (OR = 0.77, *p* = 0.019), and marital status with widowed (OR = 0.31, *p* = 0.005) were significantly associated with sarcopenia. After eight steps of eliminating the least significant independent variable, age (*p* = 0.002), sex (*p* = 0.000), marital status with widowed (*p* = 0.001), water intake (*p* < 0.018), dairy product intake (*p* < 0.019), and BMI (*p* = 0.005) were found to be indispensable predictors of sarcopenia. The logistic regression model with these six indispensable variables had a predictive value of 75.8%. Longitudinal analyses are warranted to examine whether eating behavior is a risk factor for sarcopenia onset.

## 1. Introduction

Eating a balanced and varied diet and establishing healthy eating habits promote older people’s health development across the life course; Gardner et al. concluded that “habit alone can explain about 20% of the variation in nutrition-related behaviors” [1]. Nutrition is an important determinant of the quality of life during aging that can prolong the healthy years of later life [2]. It is unclear what impact these eating behavior changes may have on frailty with sarcopenia status in rural community-dwelling elderlies. According to the statistics of the Ministry of Health and Welfare of Taiwan, the intake of dairy products, vegetables, fruits, oils, and nuts is less than the daily recommended amounts in both men and women aged ≥65 years [3]. Pan and Wu [4] also found the consumption of oils, nuts, dairy products, fruits, and vegetables in lunch to be less than the daily recommended amounts at community care centers in Taiwan. Elderly individuals aged ≥65 years accounted for 16.91% of Taiwan’s population by the end of January 2022, with the rural elderly population being the highest in Chiayi County at 21.18% [5]. Many inequalities in eating behaviors exist, with older people from households in agricultural counties generally more likely to report a poorer diet [3]. Therefore, it will be even more critical to assess the relationship between eating behavior, nutritional risk, and frailty with sarcopenia in older adults living in rural communities.

The eating behavior of the elderly population is easily affected by their living environment. With an increase in age, the chewing and swallowing abilities of elderly people change, thereby affecting their choice of food, appetite, and digestion and ultimately increasing the risk of nutritional deficiencies and frailty [4,6]. A Japanese study on community residents reported that the intake of a variety of foods may have lifespan-extending effects [7]. The intake of a variety of foods is also a protective factor against cognitive decline in the elderly population [8]. A higher intake of fruits and vegetables is associated with a significantly lower risk of cardiovascular disease and death [9,10]. A systematic review revealed that nutrition is a potential factor related to frailty in the elderly population. For instance, milk and other dairy products can help prevent physical function and cognitive impairments. In particular, the intake of more milk and yogurt provides rich milk proteins that can improve skeletal muscle mass and reduce the risk of sarcopenia among the elderly population [11].

In the community-dwelling elderly population, an unbalanced diet coupled with a lack of interaction with the community may lead to reduced dietary intake, reduced intake of different types of food, and poor nutritional status [12]. Norazman et al. [13] reported that elderly people with frailty are generally at risk of malnutrition. Frailty is a syndrome of physical decline characterized by at least two of the following symptoms on the Study of Osteoporotic Fractures (SOF) index: weight loss, exhaustion, and low mobility [14]. Poor nutrition in elderly people may not only reduce their self-care ability but also affect their immunity, quality of life, and risk of death [15]. Systematic research has also revealed that poor nutritional status is associated with the occurrence of frailty and that 15% of the community-dwelling elderly people with malnutrition have frailty [16].

Sarcopenia is a progressive and systemic skeletal muscle disease that causes accelerated loss of muscle mass and function. Elderly persons with severe sarcopenia have greater risks of falls, physical function impairments, frailty, and mortality [17]. The risk of sarcopenia or frailty can be reduced through a balanced diet, protein supplementation, and physical activity in elderly people [18]. In a previous study, the prevalence of sarcopenia was found to be 7.3% in Taiwanese adults aged ≥65 years [19]. As per the statistics of the Ministry of Health and Welfare of Taiwan, in the past year, 7.65% of the elderly people aged ≥65 years did not deliberately control their body weight but still lost more than 5% of body weight. Moreover, 16.41% of the elderly could not change their position from sitting to standing by themselves without using their hands. Furthermore, 14.38% of the elderly were in the pre-frailty stage and 17.52% were in the frailty stage [20].

Frailty and sarcopenia are key stages in disability [21]. Aging, frailty, and the risk of net weight loss are highly correlated. Many sociodemographic, behavioral, and disease-related factors are associated with sarcopenia in the community-dwelling elderly population [22]. Inadequate dietary protein intake is a predisposing factor for sarcopenia that can lead to a decrease in muscle mass with age [23]. Eating behavior and nutrition play an important role in the physical function of elderly people and may help prevent frailty and sarcopenia. Previous studies have rarely focused on eating behaviors, nutritional risks, frailty, and sarcopenia among elderly people living in rural communities and rural counties. This cross-sectional observational study aimed to assess the association of eating behavior, nutritional risk, and frailty with sarcopenia among elderly individuals living in rural communities.

## 2. Materials and Methods

### 2.1. Study Design and Population

This cross-sectional study was conducted from August 2021 to January 2022. Participants were recruited through the persons in charge of community care centers in the rural community of Chiayi County. The inclusion criteria were as follows: people aged ≥65 years who could walk independently, had no complaints of joint problems and rheumatic diseases, could converse in Mandarin and Taiwanese, and agreed to participate in this study. People who were blind, deaf, mentally ill, unable to stand, or diagnosed with moderate-to-severe cognitive impairment were excluded. In total, 208 older adults were selected through convenience sampling from 9 communities. The investigator was trained in interviews and physiological examinations, and data were collected through a structured questionnaire. The investigator conducted 20–30 min of face-to-face interviews and physiological assessments of community-dwelling elderly people. All 208 individuals who were approached consented to participate in this study. The study complied with the Declaration of Helsinki and was approved by the Chang Gung Memorial Hospital Research Ethics Review Committee (Approval Number: 202100412B0).

### 2.2. Sample Size Description

The study outcomes included the prevalence of sarcopenia, comparison of sarcopenia levels between robust participants and those with sarcopenia, and identification of factors associated with sarcopenia. As of 2022, Chiayi County has 18 towns and cities, which are major agricultural areas. The total population of this county is 499,481, with the proportion of elderly individuals being 21.1%. The sample size to assess the prevalence of sarcopenia was calculated based on Zhu’s study [19], in which the prevalence was 3.9%. Due to funding limitations, all eligible participants with sarcopenia could not be examined. Therefore, the sample size was calculated based on a confidence interval (CI) of 95%, a significance level of 0.01 in bilateral hypothesis testing, a power of 95%, and the predictor counts of 13. The total sample size for hypothesis testing was 208 participants in this study (102 robust individuals and 106 individuals with sarcopenia).

### 2.3. Participant Characteristics

Data were obtained through a self-administered structured questionnaire that assessed the following characteristics: age, sex (female or male), body mass index (BMI, kg/m^2^), marital status with widowed, presence or absence of chronic disease, medicine use, water intake, and dietary intake. BMI was calculated from the measured body weight and height. The participants were asked whether they had a partner and any chronic disease (heart disease, hypertension, diabetes mellitus, or kidney disease). Moreover, they were asked whether they used medicines and whether their water intake was at least 2000 mL per day. The participants answered “yes” or “no” to these questions. The participants’ dietary intake was assessed according to the 2018 Dietary Guidelines of the National Health Administration of the Ministry of Health and Welfare of Taiwan [23]. Six categories of food intake are recommended for adults with slightly lower activity intensity: dairy products (1.5 servings per day, 240 mL per serving); fruits (two servings per day, 100 g per serving); vegetables (three servings per day, 100 g per serving); oils and nuts (four serving per day, 5 g oil or 10 g nuts per serving); fish, eggs, and meat (four servings per day, 30–35 g per serving); and whole grains (2.5 household rice bowl per day, one bowl per serving) [24].

### 2.4. Malnutrition Universal Screening Tool

The Malnutrition Universal Screening Tool (MUST) has been used to assess nutritional risk in community-dwelling adults with high reliability [25]. The MUST uses three independent criteria to determine the overall risk of malnutrition: (1) BMI (kg/m^2^) > 20.0 is scored 0, from 18.5 to 20.0 is scored 1, and <18.5 is scored 2; (2) unplanned weight loss in the past 3–6 months <5% is scored 0, from 5% to 10% is scored 1, >10% is scored 2; and (3) a patient being acutely ill and having or being likely to have no nutritional intake for more than 5 days is scored 2. The overall risk of malnutrition is considered low (score = 0), medium (score = 1), or high (score ≥ 2).

### 2.5. SOF Index

The Study of Osteoporotic Fractures (SOF) index uses three independent criteria to determine the overall frailty: (1) weight loss ≥5% during the preceding year (regardless of any intention to lose weight), (2) inability to rise from a chair five times without using one’s arms, and (3) the answer “no” to the question “Do you feel full of energy?” The participants meeting any one criterion were considered to be in the pre-frailty stage. Frailty was defined as the presence of two or more criteria in any participant [26].

### 2.6. Sarcopenia Measurement

Sarcopenia was assessed by measuring the following: gait speed (GS), grip strength, and skeletal muscle mass (ASM) [27]. GS is the fundamental walking measure that defines a person’s basic walking ability. In this study, the participants were requested to walk for 6 m at their usual pace. Slow GS was defined as a GS <0.8 m/s, as per the 2019 recommendations of the Asian Working Group for Sarcopenia [27]. Grip strength (kg) was measured using a digital hand grip dynamometer (Baseline^®®^ Smedley Spring 12-0286, White Plains, NY, USA). The grip strength of each hand was assessed twice; the maximal value for each hand was averaged as the final estimate of hand grip strength. The grip strength was less than 28 kg for men and less than 18 kg for women, confirming a lack of muscle strength. ASM was measured using a standing posture bioelectrical impedance analysis analyzer (BC-1100-F, Tanita, Tokyo, Japan) that was already validated by Vasold et al. [28] and Yamada et al. [29]. The muscle mass index was ≤7.0 kg/m^2^ for men and ≤5.7 kg/m^2^ for women, indicating low muscle mass [27]. In general, in people aged ≥65 years, the cut-off points of the sarcopenia variables were as follows: ASM/ht^2^ (muscle mass), 5.28–5.7 kg/m^2^ in women, and 6.76–7.09 kg/m^2^ in men; gait speed (physical performance), 0.57 m/s (height ≤ 152 cm)–0.67 m/s (height > 152 cm) in women, and 0.67 m/s (height ≤ 163 cm)–0.71 m/s (height > 163 cm) in men; and handgrip strength (muscle strength), 14.6 kg and 16.4 kg (BMI < 22.3 kg/m^2^ and >26.8 kg/m^2^) in women, and 25 kg and 27.2 kg (BMI < 22.1 kg/m^2^ and >26.3 kg/m^2^) in men [30].

### 2.7. Statistical Analysis

SPSS 20.0 software (IBM Corp, Armonk, NY, USA) was used for data analysis in this study. Descriptive statistics were used to divide the participants into two groups based on their degree of sarcopenia (i.e., robust and sarcopenia). Continuous variables are presented as the means (and standard deviations [SDs]), whereas categorical variables are presented as the numbers (and percentages). The chi-squared test was performed to determine differences in the distribution of categorical variables between the groups. The Kruskal–Wallis test was performed when the homogeneity of variance was not met in an analysis of variance (ANOVA). Levene’s test was performed to assess the homogeneity of variances. ANOVA with Welch’s test was performed for unequal sample sizes. The Games–Howell post hoc test was performed when the assumption of homogeneity of variances was violated. Logistic regression analysis was performed to calculate the odds ratios (ORs) of the association of eating behavior, nutritional risk, and frailty with sarcopenia and to adjust the age and sex of the studied variables. The occurrence of an event was recorded as sarcopenia and robust, followed by further observation of key factors contributing to sarcopenia. Finally, backward stepwise logistic regression analysis was performed, resulting in a final model containing only statistically significant predictors of acceptance. The explanatory independent variables in the full model included water intake, dairy product intake, fruit intake, vegetable intake, oil and nut intake, whole grain intake, BMI, body fat content, body water percentage, frailty level, marital status with widowed, sex, and age. Values of *p* ≤ 0.05 were considered statistically significant.

## 3. Results

In total, 208 community-dwelling elders aged ≥65 years were included in this study. Most participants were female (*n* = 163, 78.4%), did have marital status with widowed (*n* = 118, 56.7%), had some chronic disease (*n* = 170, 81.7%), and had hypertension (*n* = 118, 56.7%). The robust group included 102 participants (49%); 9.76% of these had a moderate-to-high nutritional risk. The sarcopenia group included 106 participants (51%); 20.8% of these had a moderate-to-high nutritional risk. Significant differences were noted between the robust and sarcopenia groups (*p* < 0.01), regarding the participants’ sex, marital status with widowed, nutritional risk, and frailty level. Although the sarcopenia group had a higher incidence of heart disease, diabetes mellitus, and kidney disease than the robust group, the difference was not significant between the two groups (Table 1).

The average ages of the participants in the robust and sarcopenia groups were 74.42 and 79.14 years, respectively. The age (*p* = 0.001), BMI (*p* = 0.001), water intake (*p* = 0.007), dairy product intake (*p* = 0.028), fruit intake (*p* = 0.028), vegetable intake (*p* = 0.020), oil and nut intake (*p* = 0.014), whole grain intake (*p* = 0.033), gait speed (*p* = 0.001), handgrip strength (*p* = 0.001), and body fat content (*p* = 0.001) significantly differed between the robust and sarcopenia groups. The participants in the sarcopenia group had a lower water intake, BMI, average daily intake of the aforementioned types of food (except for whole grains), gait speed, handgrip strength, muscle mass, and body fat content than those in the robust group (Table 2).

**Figure 1:**
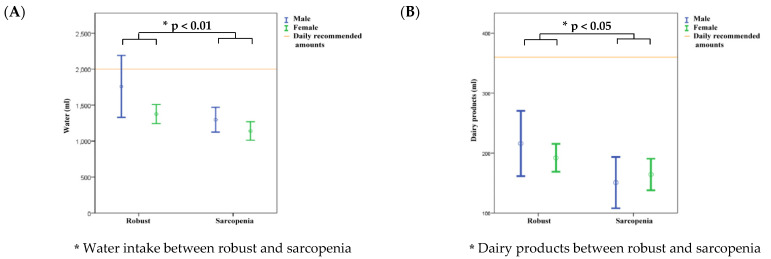

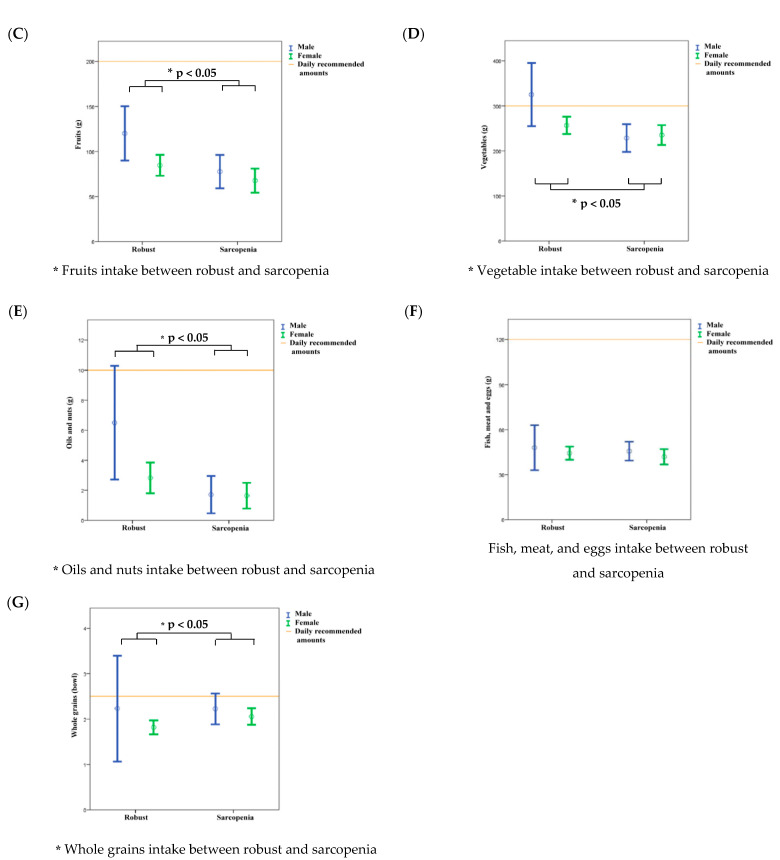
Comparison of water intake (**A**); dairy products (**B**); fruits (**C**); vegetables (**D**); oils and nuts (**E**); fish, meat and eggs (**F**), and whole grains (**G**) between participants in the robust and sarcopenia groups.

The average intake of dairy products, fruits, fish, eggs, and meat was lower among male and female participants in the robust and sarcopenia groups than among those of the daily recommended amounts (Figure 1B,C,F). A further comparison revealed that males had a higher water intake than females in both groups (Figure 1A). Both male and female participants in the robust group had a higher intake of water, fruits, vegetables, oils and nuts, fish, eggs, and meat than those in the sarcopenia group (Figure 1A–E). In the sarcopenia group, the average intake of water, fruits, oils and nuts, fish, eggs, meat, and whole grains was higher among male participants (Figure 1A,C,E–G). Moreover, the average intake (g) of dairy products and vegetables was higher among female participants in the sarcopenia group (Figure 1B,D).

Logistic regression analysis revealed that in the crude model, sarcopenia could be predicted by water intake (OR = 0.99, 95% CI = 0.99–1; *p* = 0.020), dairy product intake (OR = 0.44, 95% CI = 0.2–0.97; *p* = 0.042), body fat content (OR = 0.92, 95% CI = 0.78–0.98; *p* = 0.011), and marital status with widowed (OR = 0.37, 95% CI = 0.17–0.77; *p* = 0.008). In the adjusted model, sarcopenia was significantly associated with water intake (OR = 0.99, 95% CI = 0.99–1; *p* = 0.044), dairy product intake (OR = 0.42, 95% CI = 0.18–1; *p* = 0.049), marital status with widowed (OR = 0.31, 95% CI = 0.14–0.69; *p* = 0.005), and BMI (OR = 0.77, 95% CI = 0.62–0.96; *p* = 0.019) (Table 3).

A regression model was constructed in eight steps, reducing the original 13 variables to six (Table 4). The model with these six indispensable variables had a predictive value of 75.8%. The six predictors of sarcopenia identified in this study were as follows: age (*p* = 0.002), sex (*p* = 0.000), water intake (*p* = 0.018), dairy product intake (*p* = 0.019), BMI (*p* = 0.005), and marital status with widowed (*p* = 0.001). Thus, older men who have insufficient water and dairy product intake, have low BMI, and have no partner are generally more likely to have sarcopenia than those without these factors.

## 4. Discussion

This study explored the association of eating behavior, nutritional risk, and frailty with sarcopenia in rural community-dwelling older adults in Taiwan. Six predictors of sarcopenia were identified: age, sex, marital status with widowed, water intake, dairy product intake, and BMI. The prevalence of sarcopenia was considered to vary with sex, age, pathological conditions, and diagnostic criteria [31]. In this study, the women’s participation rate is higher than that of men. Therefore, sex as a predictor of sarcopenia requires ongoing longitudinal studies in the future. In this study, the mean age of participants in the sarcopenia group was 79.1 years, whereas that of participants in the robust group was 74.72 years. In the Foundation for the National Institutes of Health (FNIH) study, the mean age of men with sarcopenia was 70.5 years, and that of women with sarcopenia was 71.6 years [31]. The proportion of the aging population is a serious problem in rural communities of Taiwan; therefore, it is necessary to pay attention to the impact of aging on the occurrence of sarcopenia. The prevalence of sarcopenia increases with age; 10% of people aged 60–70 years have sarcopenia, and by the age of 80 years, this proportion may be nearly 30% [32].

In this study, the participants in the robust group drank more water; consumed more dairy products; and ate more fruits, vegetables, and oil and nuts than those in the sarcopenia group. The intake of water, fruits, fish, meat, and eggs was lower among the female participants than among the male participants. In addition, the female participants had a higher prevalence of sarcopenia than the male participants. A study on water intake revealed that age (incidence rate ratio (IRR) = 1.112) was an important predictor of water intake in men, whereas age (IRR = 1.103), region of residence (IRR = 0.907), and marital status (IRR = 1.051) were important predictors of water intake in women [33]. The dietary diversity score was found to be a significant contributor to the risk of sarcopenia [34]. One study revealed that a high intake of meat, fish, egg, legume, and vegetable food groups and several nutrients was associated with a lower prevalence of sarcopenia in the Korean elderly population [35]. A higher intake of fruits and vegetables may have protective effects; however, only 32% of men and women aged 65–74 years and 19% of those aged over 75 years were found to meet the recommendations of consuming five or more portions of fruits and vegetables per day in a UK Government study [36].

In this study, most older people in the sarcopenia group consumed less than the daily recommended amount of six types of food, except for whole grains. As per a study in Taiwan, among health behaviors, having an unbalanced food selection (not focusing on the six types of food), not meeting the physical activity recommendations (<150 min/week), and having a higher sitting time (≥7 h/day) were risk factors associated with sarcopenia among older adults [37]. Dietary diversity is an indicator of nutrient intake and is related to healthy aging [38]. A higher educational level, wealth index, and living in an urban area had a positive association with dietary diversity among older participants [39]. Intervention programs for sarcopenia prevention among older adults should focus on promoting balanced food selection, sufficient physical activity, and reduced sitting hours [37].

Sarcopenia was also affected by the presence or absence of a partner in our study. Mo et al. found marital status to be a significant contributor to the risk of sarcopenia [31]. In both men and women, marital status was an independent factor associated with reduced hand grip strength [40]. Therefore, more attention should be paid to the risk of sarcopenia among elderly people who live alone or those who do not have a partner.

A systematic review on the effectiveness of dairy product intake in preventing sarcopenia and frailty in the elderly population revealed some positive effects of dairy products on frailty and sarcopenia. For instance, the consumption of dairy products may reduce the risk of frailty in older adults through the addition of nutrient-rich foods to their diet [11]. In our study, dairy product intake was also an important predictor of sarcopenia. However, a narrative review based on evidence from three observational studies and eight interventional studies that used milk or fortified milk did not report any beneficial effects of milk on muscle health in older adults. This could be attributed to different habitual protein intakes, types of milk, and timings of milk consumption in different study populations, in addition to differences in the sarcopenia status of participants in the trials [41].

BMI in the robust group was significantly higher than that in the sarcopenia group in this study. Elderly persons are at an increased risk of developing sarcopenia and sarcopenic obesity characterized by an accelerated decrease in lean muscle mass and an increase in body fat [42]. In a previous study, BMI was found to be a significant contributor to the risk of sarcopenia [34]. Another study also revealed height and weight to be associated with hand grip strength [43]. In this study, the gait speed was significantly faster in the robust group than that in the sarcopenia group; however, there was no significant difference between males and females. The gait speed is presented as a physical performance. In a study that recruited 2867 community-dwelling older adults, the results reported that elderly people with gait speed cut-off points of the sarcopenia variables were as follows: 0.57 m/s (height ≤ 152 cm)–0.67 m/s (height > 152 cm) in women, and 0.67 m/s (height ≤ 163 cm)–0.71 m/s (height > 163 cm) in men [30]. In this study, women with a height of more than 153 cm (*n* = 123) and men with a height of more than 163 cm (*n* = 28) had higher abnormal rates of gait speed, 43.91%, and 85.27%, respectively.

Grip strength is another indicator used to detect sarcopenia; the average hand grip strength in elderly persons aged 80–85 years was 17.97 kg, and that in elderly persons aged over 85 years was 16.68 kg [40]. Grip strength in the robust group was significantly higher than that in the sarcopenia group in this study, and there was a significant difference between males and females. Sarcopenia is diagnosed in older adults with low muscle mass plus poor physical performance or muscle strength. Muscle mass was not significantly different between the robust and sarcopenia groups in this study; however, there was a significant difference between males and females. The muscle mass is fundamentally correlated with body size; the ASM, a sum of the muscle mass of both arms and legs, is generally used for the skeletal muscle mass index [44]. A systematic review and meta-analysis of the prevalence of sarcopenia in the world reported that overall estimates of prevalence were 10% in men and women, and the prevalence was higher among non-Asian than Asian individuals in both genders, especially when the BIA was used to measure muscle mass (19% vs. 10% in men; 20% vs. 11% in women) [45]. The prevalence of sarcopenia was significantly higher in urban women than in the rural group (5.7 versus 0.7%, respectively) in Brazil, and the environment of the women’s residence remained independently associated with sarcopenia [46].

Nutritional risk was higher in the sarcopenia group than in the robust group in our study. Malnutrition plays a key role in the pathogenesis of frailty and sarcopenia. Nutritional screening is important to identify high-risk individuals and to facilitate care management planning. A review focusing on selenium, magnesium, and omega-3 fatty acids as supplements in clinical trials and dietary interventions reported a potential association of these supplements with physical activity and muscle performance in older individuals. Muscle health is an important indicator of the functionality and independence of older adults, and certain nutrients and dietary patterns can offer protective effects against age-related reductions in strength and function [47].

Frailty and sarcopenia are important concepts to be considered for preventing physical dependence as geriatric research is shifting toward the identification of early stages of disability. Moreover, frailty has been reported to be a significant predictor of mortality (hazard ratio = 2.03, *p* = 0.001) [48]. Frailty associations were noted between dietary habits and frailty in rural Japanese community-dwelling older adults [49]. A systematic review assessing the association of sarcopenia with falls and fractures among older adults revealed that individuals with sarcopenia had a significantly higher risk of falls and fractures than those without sarcopenia [50].

This study had three limitations. First, this was a cross-sectional study, which precluded any meaningful discussions of causality. Longitudinal and interventional studies are warranted to clarify the causal relationships in the future. Second, the sample consisted of elderly people from rural communities; thus, the results cannot be generalized to other populations. Third, there are more women than men in this study, and 67% of females with sarcopenia in the study belonged to community-based care centers; the life expectancy for women in Taiwan exceeds the global average by 9.2 years, and researchers have estimated that sarcopenia in women progresses faster with age [51]. Moreover, the data have been self-reported by older people, who may have some selective memory.

## 5. Conclusions

Although this study has some limitations, our findings demonstrated that age, sex, marital status with widowed, water intake, dairy product intake, and BMI could predict sarcopenia. Early assessment of eating behavior and self-management of nutrition in rural community-dwelling older adults are essential to prevent frailty and sarcopenia. This can be achieved through community care center programs, such as providing education on consuming the six categories of food at community lunch events. Because the rural elderly populations mainly work in agriculture and their work time is limited, they tend to ignore the importance of the six categories of food in their diet. It is essential to encourage balanced eating by promoting the programs in a routine manner and by providing a comprehensive nutrient management plan for elderly people (e.g., providing education on self-perceived eating habits and reducing barriers to regularly adopting varied and balanced foods through the empowerment strategy). Our findings suggest that health policies should emphasize the early assessment of eating behaviors, BMI, age, marital status with widowed, frailty, and sarcopenia and that these should be regarded as critical factors for achieving healthy aging. Eating behavior may be a crucial indicator of sarcopenia onset; however, longitudinal studies are necessary to understand whether a causal relationship exists.

## Figures and Tables

**Table 1:** Characteristics of study participants stratified by sarcopenia level.

Variables	Overall (*n* = 208)	Robust (*n* = 102)	Sarcopenia (*n* = 106)	*p*-Value ^a^
Sex, female, *n* (%)	163 (78.4%)	92 (90.2%)	71 (66.98%)	0.001
Marital status (widowed), *n* (%)	118 (56.7%)	48 (47.06%)	70 (66.04%)	0.004
Chronic disease (Yes), *n* (%)	170 (81.7%)	81 (79.41%)	89 (83.96%)	0.396
Heart disease (Yes), *n* (%)	32 (15.4%)	15 (14.71%)	17 (16.04%)	0.071
Hypertension (Yes), *n* (%)	118 (56.7%)	63 (61.76%)	55 (51.89%)	0.151
Diabetes mellitus (Yes), *n* (%)	46 (22.11%)	21 (20.59%)	25 (23.58%)	0.603
Kidney disease (Yes), *n* (%)	4 (1.9%)	1 (0.98%)	3 (2.83%)	0.331
Nutritional risk (low)	176 (84.6%)	92 (90.20%)	84 (79.24%)	
Nutritional risk (medium)	25 (12%)	7 (6.86%)	18 (19.98%)	0.006
Nutritional risk (high)	7 (3.4%)	3 (2.9%)	4 (3.8%)	
Frailty level (robust)	164 (78.8%)	90 (88.24%)	74 (69.81%)	0.004
Frailty level (pre-frailty, frailty)	44 (21.2%)	12 (11.76%)	32 (30.19%)	

^a^ χ^2^ test for proportions and nominal variables.

**Table 2:** Participants’ age, BMI, intake of different types of food, and body composition characteristics stratified by sarcopenia level.

Variables	Overall (*n* = 208) M (SD)	Robust (*n* = 102) M (SD)	Sarcopenia (*n* = 106) M (SD)	*p*-Value ^a^
Age (years)	76.83 (7.29)	74.42 (6.74)	79.14 (7.07)	0.001
BMI (kg/m^2^)	24.31 (3.94)	25.36 (4.2)	23.3 (3.39)	0.001
Water (mL)	1300.96 (599.11)	1413.73 (642.71)	1192.45 (534.82)	0.007
Dairy products (s1)	0.74 (0.47)	0.81 (0.45)	0.67 (0.48)	0.028
Fruit (s2)	0.79 (0.56)	0.88 (0.56)	0.71 (0.56)	0.028
Vegetable (s3)	2.48 (0.94)	2.63 (0.95)	2.33 (0.91)	0.020
Oil, nuts (s4)	0.24 (0.94)	0.32 (0.51)	0.17 (0.36)	0.014
Fish, meat, eggs (s5)	1.46 (0.69)	1.49 (0.71)	1.44 (0.68)	0.600
Whole grains (s6)	1.99 (0.86)	1.86 (0.86)	2.11 (0.84)	0.033
Gait speed (m/s)	10.65 (4.84)	7.86 (2.53)	12.99 (5.30)	0.001
Male	11.25 (4.54)	6.57 (0.43)	12.59 (0.72)	0.155 ^b^
Female	10.08 (4.91)	8.00 (0.27)	13.23 (0.76)	
Handgrip strength (kg)	20.18 (6.55)	23.54 (5.33)	16.62 (5.82)	0.001
Male	23.74 (7.97)	34.93 (1.48)	20.55 (0.91)	0.001 ^b^
Female	19.12 (5.67)	22.28 (0.38)	14.33 (0.61)	
Muscle mass (kg)	37.31 (7.73)	37.73 (7.04)	36.89 (8.38)	0.445
Male	47.18 (5.51)	51.91 (1.87)	45.93 (0.83)	0.001 ^b^
Female	34.55 (5.76)	36.3 (0.57)	32.16 (0.67)	
Body fat (kg)	32.36 (8.60)	35.08 (7.6)	29.73 (8.73)	0.001
Male	23.1 (6.56)	25.45 (1.46)	22.43 (1.17)	0.001 ^b^
Female	34.97 (7.2)	36.14 (0.75)	33.43 (0.85)	

^a^ M (SD), mean ± standard deviation for continuous variables; CI, confidence interval; BMI, body mass index; s1, dairy products, 240 mL per serving; s2, fruits, 100 g per serving; s3, vegetables, 100 g per serving; s4, oils and nuts, 5 g oil or 10 g nuts per serving; s5, fish, meat, and eggs, 30–35 g per serving; and s6, whole grains, one bowl per serving; *p*-value obtained using the *t*-test; ^b^ comparative analysis of male and female.

**Table 3:** Relationship of types of food, marital status, BMI, body composition, and frailty level with sarcopenia.

		Crude Model			Adjusted Model	
Variables	OR	95% CI	*p*-Value	OR	95% CI	*p*-Value ^a^
Water (mL)	0.99	0.99, 1	0.020	0.99	(0.99, 1)	0.044
Dairy products (s1)	0.44	0.2, 0.97	0.042	0.42	(0.18, 1)	0.049
Fruit (s2)	0.8	0.4, 1.6	0.528	0.79	(0.38, 1.66)	0.538
Vegetable (s3)	0.92	0.62, 1.38	0.691	1.01	(0.66, 1.54)	0.973
Oil, nuts (s4)	0.71	0.32, 1.59	0.407	0.61	(0.25, 1.47)	0.271
Whole grains (s5)	1.31	0.89, 1.94	0.173	1.23	(0.81, 1.87)	0.334
Marital status with widowed	0.37	0.17, 0.77	0.008	0.31	(0.14, 0.69)	0.005
BMI	1.02	0.9, 1.16	0.784	0.77	(0.62, 0.96)	0.019
Body fat, kg	0.92	0.87, 0.98	0.011	1.06	(0.96, 1.17)	0.283
Body water, %	1.06	0.98, 1.13	0.134	0.95	(0.88, 1.04)	0.272
Frailty level	2.13	0.88, 5.18	0.094	1.58	(0.6, 4.19)	0.356

^a^ BMI, body mass index; OR, odds ratio; CI, confidence interval; s1, dairy products, 240 mL per serving; s2, fruits, 100 g per serving; s3, vegetables, 100 g per serving; s4, oils and nuts, 5 g oil or 10 g nuts per serving; and s5, whole grains, one bowl per serving. The adjusted model was adjusted for sex and age.

**Table 4:** Association of independent variables with sarcopenia.

		B	SE	*p*-Value	OR (95% CI)
Step 1	Age	0.078	0.030	0.007	1.08 (1.02, 1.14)
	Sex	−3.487	0.980	0.000	0.03 (0, 0.21)
	Water (mL)	−0.001	0.000	0.044	0.99 (0.99, 1)
	Dairy products (s1)	−0.856	0.430	0.049	0.42 (0.18, 1)
	Fruit (s2)	−0.232	0.380	0.538	0.79 (0.38, 1.66)
	Vegetable (s3)	0.007	0.220	0.973	1.01 (0.66, 1.54)
	Oil, nuts (s4)	−0.497	0.450	0.271	0.61 (0.25, 1.47)
	Whole grains (s5)	0.207	0.210	0.334	1.23 (0.81, 1.87)
	Marital status with widowed	−1.181	0.420	0.005	0.31 (0.14, 0.69)
	BMI	−0.261	0.110	0.019	0.77 (0.62, 0.96)
	Body fat, kg	0.056	0.050	0.283	1.06 (0.96, 1.17)
	Body water, %	−0.048	0.040	0.272	0.95 (0.88, 1.04)
	Frailty level	0.459	0.500	0.356	1.58 (0.6, 4.19)
Step 8	Age	0.085	0.030	0.002	1.09 (1.03, 1.15)
	Sex	−2.498	0.540	0.000	0.08 (0.03, 0.24)
	Water (mL)	−0.001	0.000	0.018	0.99 (0.99, 1)
	Dairy products (s1)	−0.994	0.420	0.019	0.37 (0.16, 0.85)
	Marital status with widowed	−1.331	0.400	0.001	0.26 (0.12, 0.58)
	BMI	−0.146	0.050	0.005	0.86 (0.78, 0.96)

BMI, body mass index; B, beta coefficient; SE, standard error; OR, odds ratio; s1, dairy products, 240 mL per serving; s2, fruits, 100 g per serving; s3, vegetables, 100 g per serving; s4, oils and nuts, 5 g oil or 10 g nuts per serving; and s5, whole grains, one bowl per serving.

## Data Availability

The data supporting the findings of the study are available from the corresponding author on reasonable request.
